# Clinician uptake of obesity-related drug information: a qualitative assessment using continuing medical education activities

**DOI:** 10.1186/1475-2891-12-44

**Published:** 2013-04-10

**Authors:** Ingrid Kohlstadt, Gerold Wharton

**Affiliations:** 1 Johns Hopkins Bloomberg School of Public Health, Center for Human Nutrition, 198 Prince George St, Annapolis MD 21401, Maryland, USA; 2 The U.S. Food and Drug Administration, Office of Pediatric Therapeutics, Silver Spring, Maryland, USA

**Keywords:** Medication effects on appetite, Insulin resistance, Drug-related weight gain, Mental illness as a risk factor for obesity, Adverse metabolic drug effects, Drug safety research, Nutrition knowledge of primary care practitioners

## Abstract

**Background:**

Medications necessary for disease management can simultaneously contribute to weight gain, especially in children. Patients with preexisting obesity are more susceptible to medication-related weight gain.

How equipped are primary care practitioners at identifying and potentially reducing medication-related weight gain? To inform this question germane to public health we sought to identify potential gaps in clinician knowledge related to metabolic adverse drug effects of weight gain.

**Methods:**

The study analyzed practitioner responses to the pre-activity questions of six continuing medical education (CME) activities from May 2009 through August 2010.

**Results:**

The 20,705 consecutive, self-selected respondents indicated varied levels of familiarity with adverse metabolic effects and psychiatric indications of atypical antipsychotics. Correct responses were lower than predicted for drug indications pertaining to autism (−17% predicted); drug effects on insulin resistance (−62% predicted); chronic disease risk in mental illness (−34% predicted); and drug safety research (−40% predicted). Pediatrician knowledge scores were similar to other primary care practitioners.

**Conclusions:**

Clinicians’ knowledge of medication-related weight gain may lead them to overestimate the benefits of a drug in relation to its metabolic risks. The knowledge base of pediatricians appears comparable to their counterparts in adult medicine, even though metabolic drug effects in children have only become prevalent recently.

## Background

No study to date assesses the knowledge base around medication-related weight gain in pediatric or adult primary care medicine. We therefore sought to characterize what practitioners know about metabolic drug effects in the context of clinical decision-making.

Informed clinicians can often modify their patients’ risk of adverse metabolic drug effects, even when medications are essential for disease management [1]. Practitioners can choose lowest effective dosing and therapies with fewer metabolic effects; treat underlying medical conditions which can contribute to weight gain, such as sleep apnea and hypothyroidism; correct nutritional deficiencies such as vitamins B_12_[2] and D [3] to facilitate lifestyle adherence; and counsel patients on drug-related increases in appetite, emphasizing adherence to medication and healthful lifestyle choices.

Among the patient groups most vulnerable to metabolic drug effects are children. Children are more susceptible to central nervous system effects of medications [4]. Some metabolic drug effects are unique to children at certain growth stages and demonstrate a prolonged effect [5,6]. Metabolic drug effects also tend to be delayed relative to the therapeutic benefit, especially in children. Concurrently, drug exposure is increasing in children, the age group with the fastest growing number of prescriptions [7], in part due to obesity-related chronic diseases. Preexisting overweight and obesity heighten vulnerability to metabolic drug effects.

Managing adverse metabolic drug affects is relatively new to the practice of pediatrics. Historically pediatricians focused on medication-related weight loss and stunting, recorded as step-offs on patient growth charts. Today’s pediatric practice may require as diligent a diagnosis and management of medication-related weight gain, especially since preexisting overweight and obesity, defined as a body mass index at or above the 85^th^ percentile, has reached approximately 32% of the U.S. population ages 2-19 [8,9].

Disseminating drug safety updates to pediatricians holds other challenges as well. Safety information specific to children represents a recent advance. Practitioners may not realize they need to watch for such updates [10]. Metabolic drug effects specific to children and adolescents may be first identified years after a drug is on the market [11] because the metabolic effects in children tend to manifest beyond the timeframe of clinical trials. Disseminating drug safety information may be additionally complicated by practice patterns. For example, psychiatrists may diagnose and prescribe highly specialized treatment and look to primary care practitioners to monitor patients for adverse drug effects.

Clinicians draw on their knowledge base of adverse metabolic drug effects for clinical decision-making. Elevated and unique risks of metabolic drug effects and major shifts in disease prevalence and practice patterns in pediatrics together prompted our interest in confirming that primary care clinicians who care for children have a knowledge base comparable to their adult medicine counterparts.

## Methods

### CME partners

Continuing medical education (CME) activities were developed in partnership with CME providers. Inclusion criteria for partners were: experience implementing pre-activity questions, having primary care practitioners as a target audience, willingness to co-develop programs relevant to medication-associated weight gain, providing free public access to associated media and print materials, and collaborating within time and budget constraints. Partners were selected across different media - audio, lectures, or web-based activities - Audio-Digest Foundation, Medscape CME, The Maryland Academy of Family Physicians, and The FDA Scientific Rounds Program.

### Instrument development

The instrument in this study, pre-CME activity questions, measures practitioners’ baseline knowledge relevant to the content of the CME activities. Pre-activity questions were 4-choice multiple choice questions or true-false questions. They were directed at clinical decision-making and were organized into four categories: 1) drug indications, 2) metabolic drug effects, 3) drug safety updates, and 4) patients most at risk. Each CME program partner selected among the pre-activity questions and adapted the wording to their standard format.

### CME content

The 6 CME activities pertained to either atypical antipsychotic use in children or obesogenic medications in general. They were provided through the CME partners at varied intervals between June 2009 and August 2010. Each activity was an audio program, web-based program, or conference lecture; and awarded a maximum of 0.5 to 2 Category 1 CME credits. The program content, participant characteristics, and pre-activity questions are presented in Table 1.

**Table 1:** Summary of continuing medical education (CME) programs, June 2009 – August 2010

**CME partner and activity**	**Date, audience and promotions**	**Pre-activity questions**
Activity 1: Maryland Academy of Family Physicians, 2009 Annual CME Assembly; Drug –nutrient interactions	6/09 Conference attendees, primarily family physicians in the mid-Atlantic	5 interspersed questions using an audience response system
Activity 2: Audio-Digest Pediatrics on Atypical antipsychotics in children	8/09-8/10 Subscribers, practitioners who care for children, approximately 75% physicians	10 questions submitted by mail, fax, or website
Activity 3: Audio-Digest Special Topics on Atypical antipsychotics in children *	8/09-8/10 Responders to free CME post on the Audio-Digest homepage; American Academy of Pediatrics newsletter feature	10 questions submitted via website
Activity 4: Medscape CME on Atypical antipsychotics in children *	11/09-1/10 Participants in Medscape’s Psychiatry and Mental Health listserv with multiple cross-posts	6 web-based interspersed questions
Activity 5: Medscape CME on Medication-related weight gain *	5/10-8/10 Participants in Medscape’s Preventive Medicine and Public Health listserv with multiple cross-posts; American College of Preventive Medicine feature	4 web-based interspersed questions
Activity 6: Food and Drug Administration Scientific Rounds on Autism	5/10 FDA employees with regulatory and clinical interests	3 questions using an audience response system, 1 which was developed to better understand responses from prior CME programs

*Three of the activities may be viewed online [12-14].

In order to compare the knowledge of practitioners specializing in pediatrics with adult medicine practitioners, we developed Activity 5 which is applicable to the care of children and adults.

Activity 6 was the only program where the target audience was not primary care practitioners. The biweekly activity is attended by a diverse group of health care practitioners and scientists, all of whom work in regulation. The activity was included to better characterize practitioner knowledge of the autism indication for atypical antipsychotics.

### Response analysis

The information used for the response analysis was obtained from the CME providers as anonymized source data with no way to match responses with individuals. No personal identifiers were used. The respondents were participating in CME activities, where responses to the related learning questions are routinely aggregated to inform future CME development and related research.

We analyzed the data with and without comparing it to predicted scores. Predicted scores facilitate comparison between multiple choice questions with four choices and binomial true-false questions, which differ in the likelihood of selecting a correct answer by chance alone. For this analysis, predicted scores were 70% for multiple choice and 85% for true-false questions. The basis for these numbers comes from Audio-Digest’s overall average pretest scores, which are 70%, [personal communication August 2010] and the pedagogic intent of a CME to build on participants’ existing practice-relevant knowledge.

Each response holds an inherent error, since a participant with a constant knowledge base could score better or worse on the pre-activity depending on the circumstances at that moment. We estimated the two-tailed two standard deviations of this variability to be ten percent. We also analyzed the participants’ responses to the choice identified as close to correct, also called the second best answer.

STATA® statistical software was used to run discrete-response regression analyses on pre-activity question responses. Probit regressions were used for binomial dependent variables, analyzing whether the respondent answered the CME question correctly. The probit models give a standard normal z-score rather than a t-statistic, with the total variability explained as a pseudo-R^2^ rather than a normal R^2^. McFadden’s pseudo-R^2^ is reported. The probit analysis reports the overall significance of the model using an LR chi-square. The effect of a control variable in predicting correct responses (a certain percentage above/below the average) is calculated as the difference in probability of getting a question correct versus a baseline probability. For this analysis, baseline probability is where all control variables are set to their population means.

The control variables used in the probit models were educational degree, medical specialty, and CME participation date. Geographic region was only provided by some respondents and was therefore not included in the analysis. The type of medical practice such as hospital-based or solo practice was not among the data obtained by the CME providers.

Partial incomplete responses were included in the analysis. Having all pre-activity questions left blank was considered equivalent to nonparticipation, and these entries were excluded. Since the sample size of the distinct CME activities varied, both the unweighted and weighted averages of correct responses are reported.

### Instrument validation

To assess the extent to which the pre-activity responses could be generalized among primary care practitioners, the responses were compared across the diverse CME programs detailed in Table 1.

The scores on pre-activity responses were compared to self-reported learning in Activities 2–3, where participants were asked, “Please list one concept or strategy gained from this activity.”

Participant evaluations of the CME programs were recorded, to confirm satisfactory evaluations. The rating of the CME activity on a 1–5 Likert scale is a composite score which reflects practice relevance and appropriate teaching level of target population.

Not all participants completed all pre-activity questions. The data was analyzed both including and excluding question-specific non-responders, to detect a potential bias introduced by partial completion.

Activities 2 and 3 were the longest-running programs, each offered for 13 months. They were analyzed for a temporal trend, since a news story or regulatory change during the interval could potentially change practitioner baseline knowledge or practice patterns.

## Results

There were 20,705 participants in the combined six CME activities which spanned 15 months. Each participant answered one or more of the following questions.

### Drug indications

See Table 2. For the first question, both the average correct response rate of 76% and the weighted correct response of 79% are within the predicted range. For the second question, the average correct response rate is 53% (17% below predicted) and the weighted average correct response rate is 52% (18% below predicted).

**Table 2:** Responses to multiple choice pre-activity questions on use of antipsychotic medications

**CME activity**	**Activity 2**	**Activity 3**	**Activity 4**
Sample size	*n*=1237	*n*=611	*n*=2400
Responses to: Which of the following is a labeled indication for one or more atypical antipsychotic drugs?			
A. Refractory epilepsy			
B. Refractory major depression in adolescents			
**C. Acute mania associated with bipolar-I disorder**			
D. Attention deficit/hyperactivity disorder			
Incorrect (A)	82 (7%)	40 (6%)	72 (3%)
Incorrect (B)	188 (15%)	92 (15%)	264 (11%)
**Correct (C)**	**912 (74%)**	**439 (72%)**	**1992 (83%)**
Incorrect (D)	55 (4%)	40 (6%)	72 (3%)
Correct vs. Predicted	+4%	+2%	+13%
Responses to: Which of the following is a labeled indication for an atypical antipsychotic in a child, 7 years of age?			
**A. Irritability associated with autism**			
B. Acute bipolar mania			
C. Schizophrenia			
D. Generalized anxiety disorder			
**Correct (A)**	**777 (63%)**	**288 (47%)**	**1152 (48%)**
Incorrect (B)	197 (16%)	114 (19%)	456 (19%)
Incorrect (C)	222 (18%)	189 (31%)	720 (30%)
Incorrect (D)	38 (3%)	19 (3%)	72 (3%)
Correct vs. Predicted	−7%	−23%	−22%

See Table 3. The average correct response rate is 65% (20% below predicted) and the weighted average is 67% (18% below predicted).

**Table 3:** Responses to the true-false pre-activity question on use of antipsychotic medications

**CME activity**	**Activity 2**	**Activity 3**
Sample size	*n*=1237	*n*=611
Responses to: The intramuscular administration of at least one atypical antipsychotic agent has been approved by the FDA for use in children. True or false?		
Incorrect (True)	341 (28%)	265 (43%)
**Correct (False)**	**896 (72%)**	**346 (57%)**
Correct vs. Predicted	−13%	−28%

The participants in Activity 6 were asked: Recommended treatment of autism includes all EXCEPT:

A Correct nutritional deficiencies 6 (12%)

B Treatment of concurrent attention-deficit hyperactivity disorder 5 (10%)

C Prescribe atypical antipsychotics 29 (57%)

D Use behavioral therapies following early diagnosis 11 (21%)

The rate of correct response, response C, is 57% (13% below predicted).

### Adverse metabolic effects

Participants in Activity 5 were asked to respond to: After diagnosing Ed with metabolic syndrome, Ed’s doctor advised him to reduce his weight by 10%, a total of 18 pounds, by diet and exercise. Which of the following medications potentially makes it more difficult for Ed to achieve his goal?

A Angiotensin-converting enzyme inhibitors 6667 (41%)

B Diuretic 1243 (8%)

C Vitamin D 1106 (7%)

D Biguanide 7021 (43%)

The rate of correct response, choice B, is 8% (62% below predicted). Specialty did not predict response to this question.

Participants in Activity 5 were also asked to respond to: Within months of being diagnosed with bipolar disorder at age 14, Sara gained 20 pounds. Which of the following is likely to contribute to her recent weight gain and body mass index of 28?

A Vitamin D deficiency 952 (6%)

B Atypical antipsychotic agent 10349 (63%)

C An eating disorder 1072 (7%)

D Psychostimulant agent 3948 (24%)

The average correct response rate of 63% falls within the predicted range. Predicted probability of answering the question correctly given the regression control variables is 65%. Analysis by specialty indicates that mental health specialists scored 28% better than average (z=27; p<0.01), family practitioners scored 14% higher (z=12; p<0.01), internal medicine specialists scored 9% higher (z=7; p<0.01), endocrinologists scored 8% higher (z=3; p<0.01). The regression explains 9% (pseudo-R^2^=0.09) of the total variability in responses and was very significant in predicting scores (LR chi-square=1833; p<0.01).

Table 4 indicates the responses to a pre-activity question on adverse drug effects. See Table 5. For the first question, the average correct response rate was 61% with a weighted correct response average of 67%. For the second question, the average correct response rate was 75% with a weighted average also of 75%. These are within the predicted range.

**Table 4:** Responses to the true-false pre-activity question on adverse drug effects

**CME activity**	**Activity 2**	**Activity 3**
Sample size	*n*=1237	*n*=611
Responses to: Hyperglycemia, hyperlipidemia, and elevated liver enzymes are labeled side effects for one or more atypical antipsychotics. True or false?		
**Correct (True)**	**1154 (93%)**	**582 (95%)**
Incorrect (False)	83 (7%)	29 (5%)
Correct vs. Predicted	+8%	+10%

**Table 5:** Responses to multiple choice pre-activity questions on adverse drug effects

**CME activity**	**Activity 1**	**Activity 2**	**Activity 3**	**Activity 4**
Sample size	*n*=39	*n*=1237	*n*=611	*n*=2400
Responses to: Jeff is an athletic 14-year-old who has been diagnosed with schizophrenia and prescribed an atypical antipsychotic. You counsel the family about potential cardio-metabolic side effects. Which risk would you emphasize?				
A. Sudden death from myocardial infarction				
B. Cardiac arrhythmias				
**C. Increases in triglycerides, total cholesterol, and low-density lipoprotein**				
D. Because patient has normal weight, he is unlikely to experience significant weight gain				
Incorrect (A)	n/a	88 (7%)	34 (6%)	120 (5%)
Incorrect (B)	n/a	173 (14%)	132 (22%)	408 (17%)
**Correct (C)**	**n/a**	**726 (59%)**	**288 (47%)**	**1848 (77%)**
Incorrect (D)	n/a	245 (20%)	156 (25%)	48 (2%)
Correct vs. Predicted	n/a	−11%	−23%	+7%
Responses to: Three months after initiating treatment with antipsychotic medication, Jeff has blood-work to monitor lipids, liver enzymes, and glucose. A work-up for what endocrine condition may additionally be indicated?				
A. Hypothyroidism				
B. **Hyperprolactinemia**				
C. Hyperparathyroidism				
D. Hyperthyroidism				
Incorrect (A)	4 (10%)	144 (12%)	101 (17%)	432 (18%)
**Correct (B)**	**27 (75%)**	**974 (79%)**	**443 (73%)**	**1752 (73%)**
Incorrect (C)	3 (8%)	57 (5%)	22 (3%)	72 (3%)
Incorrect (D)	5 (13%)	55 (4%)	44 (7%)	144 (6%)
Correct vs. Predicted	+5%	+9%	+3%	+3%

### Patients at increased risk

Responses to a question about vulnerable populations are presented in Table 6. The average correct response rate is 36% (34% below predicted) with a weighted average of 33% (37% below predicted).

**Table 6:** Responses to the pre-activity question on vulnerable populations

**CME activity**	**Activity 1**	**Activity 2**	**Activity 3**	**Activity 4**	**Activity 5**
Sample size	*n*=45	*n*=1237	*N*=611	*n*=2400	*n*=16361
Responses to: Mental illness shortens lifespan as follows:					
**A. Patients with mental illness die 25 years earlier than the general population, mostly due to earlier onset of chronic medical conditions.**					
B. The average 15 years of potential life lost can be explained by premature death early in life.					
C. The causes of mortality are similar to the general population, but occur 10 years earlier on average.					
D. Suicide and accidents including motor vehicle accidents account for most of the 10 years of shorter life expectancy.					
**Correct (A)**	**13 (29%)**	**629 (51%)**	**215 (35%)**	**840 (35%)**	**5166 (32%)**
Incorrect (B)	9 (20%)	285 (23%)	184 (30%)	144 (6%)	1584 (10%)
Next best answer (C)	20 (44%)	265 (21%)	169 (28%)	528 (22%)	4557 (28%)
Incorrect (D)	3 (7%)	55 (5%)	42 (7%)	888 (37%)	4718 (29%)
Correct vs. Predicted	−41%	−19%	−35%	−35%	−38%

**Figure 1:**
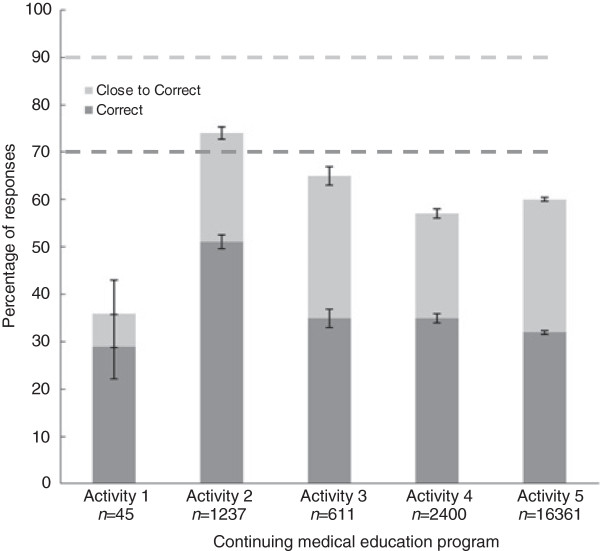
Responses to pre-activity question on chronic disease risk in mental illness.

Figure 1 illustrates the correct responses compared to the predicted responses for the pre-activity question on mental illness and chronic disease risk. The responses are presented across CME activities 1–5. Standard error bars are shown. Since one of the three incorrect responses (Choice C) in the question was close to the correct answer, it may reflect a stronger knowledge base than the other two incorrect choices. We therefore included this response in the figure.

Activity 5’s large sample size allowed for further analysis. Participants had a predicted probability of 31% in answering correctly. Participants specializing in mental health scored 14% higher than average (z=11; p<0.01) and family practitioners scored 4% higher (z=3; p<0.05). The probit regression explained 1% (pseudo-R^2^=0.01) of the total variability in the responses and was significant (LR chi-square=202, p<0.01).

### Drug safety updates

Participants in Activity 5 were asked to respond to: Which of the following statements is correct?

A. Comparative effectiveness trials are part of the drug approval process [5622 (34%)]

B. Phase 3 clinical trials are powered to identify appetite-stimulating effects of medication [3346 (20%)]

C. Incidence of weight gain can be calculated from a passive adverse events reporting system [4424 (27%)]

D. Current legislation requires clinical trials in pediatric populations [2452 (15%)]

On average, 15% of participants answered correctly (55% below predicted) selecting choice D. Predicted probability of answering correctly given the regression variables is 14%. Analysis by specialty indicates that pediatricians scored 7% higher than average (z=6; p<0.01) and mental health specialists scored 2% higher (z=2; p<0.02). General practitioners scored 3% below average (z=−2; p<0.03) and emergency medicine specialists scored 4% lower (z=−2; p<0.04). The regression explained 2% of the total variability in answers (pseudo-R^2^=0.02) and was very significant (LR chi-square=242; p<0.01).

Note that this question had the highest non-response rate, with 517 (3%) of participants leaving the question blank. Regression analysis excluding non-responders had the same significant outcome variables as the analysis which included non-responders.

See Table 7. The average correct response was 47% (23% below predicted) and the weighted average was 51% (19% below predicted).

**Table 7:** Responses to pre-activity questions on drug safety information

**CME activity**	**Activity 2**	**Activity 3**
Sample size	n=1237	n=611
Responses to: Practitioners are asked to report drug adverse events:		
**A. To MedWatch**		
B. Only when the adverse event is not specified on the drug label		
C. Within 30 days of occurrence, as required by law.		
D. Voluntarily, if observed within 30 days of the first dose		
**Correct (A)**	**698 (56%)**	**237 (39%)**
Incorrect (B)	85 (7%)	53 (9%)
Incorrect (C)	203 (10%)	174 (29%)
Incorrect (D)	243 (20%)	146 (24%)
Correct vs. Predicted	−14%	−31%

For Activity 5 (*n*=16,361), the top three professions of participants were nurse practitioners (52%, *n*=8407), physicians (38%, *n*=6212), and physician assistants (3%, *n*=476). The top specialties were psychiatry/mental health (12%, *n*=2022), family medicine (11%, *n*=1875), internal medicine (10%, *n*=1639), general practice (6%, *n*=946), and pediatrics (6%, *n*=906).

We controlled the regression analysis for predicting correct pre-activity responses for specialty, professional degree, and date of CME participation by quartile. The time of participation was included in the regression analysis because it explained a significant portion of the variability but yielded no clear pattern for interpretation. Results of the regression analysis follow each applicable question. The results of the analysis by specialty concur with the practice demands of each specialty. For example, family physicians, practitioners who follow patients across the lifespan, were more likely to correctly identify the profound extent to which mental illness shortens life expectancy due to chronic diseases.

### Instrument analysis

The strength of the instrument is its ease of use in the context of CME programming, and its associated ability to identify trends in practitioner knowledge and some broad comparisons among practitioners. However since the instrument is comprised of multiple choice questions, responses to any one question are more appropriately viewed in the context of the full instrument.

In order to assess the variability of the instrument, responses were compared across CME programs which varied in content, timeframe, recruitment, and question administration. Figure 1 depicts the responses. Responses among CME programs varied within the pre-established +/−10% test error, except for one program with a small sample size. The unweighted, correct response averages across CME programs are reported.

To assess the extent to which recruitment methods may influence the pre-activity responses, the overall scores of the two Audio-Digest programs were compared. The two programs differ only in how the participants were recruited. They were recruited as either, subscribers or one-time participants. The 10% difference in responses falls within the pre-established test error.

Practice-relevance and the perception of the CME program’s usefulness were considered in the instrument analysis. The participants in each of the 6 activities were asked to evaluate the program on a 1–5 Likert scale, 5 being the highest score. The ratings for each program ranged from 4.0 to 5.0, with an unweighted mean score of 4.5, suggesting that all were well-received and applicable to participants’ clinical practice.

The pre-activity question responses were correlated with what participants said they learned from the activity. Participants in Activities 2 and 3 were asked to “Please list one concept or strategy gained from this activity.” The written responses fell into categories consistent with pre-activity responses: Pediatric indications (20), patient adherence (2), adverse effects (71), MedWatch reporting (9), drug interactions (5), and patient risk factor (8).

A temporal trend in correct responses was not observed between the first three months and the total 13 months of the responses to Activities 2 and 3. Neither was any single news event or regulatory change identified which might be anticipated to influence practitioner knowledge on this topic during the study period.

## Discussion

The childhood obesity epidemic is recent; however, practitioners who care for children appear as familiar with adverse metabolic drug effects as practitioners who care for adults. Those specializing in pediatrics performed better on a question about drug research, perhaps reflecting recent educational activities directed towards pediatricians.

Across medical specialties practitioner knowledge of medication-related weight gain was low in four areas of our study. Each of the knowledge gaps if practice relevant, would overestimate the medication’s benefits or underestimate adverse metabolic effects. The net effect of each knowledge gap would therefore affect clinical decision-making in the same direction, potentially contributing to excess metabolic dysfunction. The four areas of low practitioner knowledge are as follows:

Responses to questions about drug indications and the use of antipsychotics in autism suggest that some practitioners may mistake the management of aggressive symptoms for treatment of the underlying disease process. Additionally, new oral preparations are available for children who have difficulty swallowing pills. These preparations should be used before prescribing intramuscular preparations, which have greater metabolic effects and do not have pediatric use indications at the time this manuscript is written.

Among the questions pertaining to adverse metabolic effects, only 8% of practitioners selected the intended response that some diuretics have been associated with promoting insulin resistance. The 41% who incorrectly selected “angiotensin-converting enzyme inhibitors” are unlikely to have been aware that some diuretics promote insulin resistance and angiotensin converting enzymes, in contrast, may be insulin sensitizing [15], the distinction between these two antihypertensive therapies in a patient with metabolic syndrome would be practice relevant. Furthermore, these respondents may have erroneously equated the reduction in peripheral edema with meaningful, long-term weight loss among their patients. The 43% of practitioners who incorrectly selected “biguanide” may not have realized that metformin is in this medication class, so the response would have been more informative if the answer had read “biguanide (metformin).”

Responses reflected low baseline knowledge of drug safety research and MedWatch, a passive surveillance program. It is possible that practitioners lack a framework for managing the escalating drug-related information. Our findings parallel those of a recent study of physician knowledge and adverse events reporting of dietary supplements [16].

Mental illness is associated with increased vulnerability to adverse metabolic effects. The profound chronic disease mortality among patients with mental illness was under-recognized across specialties as measured by our instrument. Awareness of the high mortality from chronic diseases among patients with mental illness might be unlikely to cause practitioners to alter the patient’s psychiatric medications; however, it would guide overall care such as screening, referring, concurrent medication prescribing, and managing co-morbidities.

The knowledge gaps parallel the few peer-reviewed publications on medication-related weight gain, other than the atypical antipsychotics. Reviewing the proceeding of a large international conference on obesity [17] revealed a similar paucity of research and translational initiatives surrounding medication-related weight gain. Additionally, current drug product information and labeling lacks a consistent format or location for communicating the potential effects of a drug on the patient’s appetite and underlying metabolism.

Practitioners were familiar with the general indications for use of atypical antipsychotics in children and the adverse metabolic effects including prolactinemia, dyslipidemia, elevated liver enzymes, insulin resistance, and weight gain, findings which correlate with the lay and medical literature’s recent attention to the topic [4]. Similarly, education initiatives about pediatric drug labeling have been directed to pediatricians and pediatricians were more knowledgeable than other practitioners about ongoing pharmacovigilance.

The instrument demonstrated internal consistency across diverse CME programs (Table 1), suggesting the findings may appropriately be generalized across U.S. primary care practitioners. The sampling frame captures participants across the United States, with diverse patient populations in diverse practice settings. The degrees of the participants, nurse practitioners, physician assistants, and medical doctors, correctly represent the educational diversity of primary care practitioners. Pediatricians scored as well as their adult medicine counterparts, suggesting that future initiatives could appropriately be directed to all primary care practitioners.

Additional merits of the instrument are that it can be implemented in a timely and cost-sensitive way. It can be applied to assess evidence-based practice knowledge [18]. Study findings can provide baseline data, by which to gauge the effectiveness of future interventions. The instrument also provides a continuing education curriculum developed free of industry interests. An internet curriculum on safe medication use measurably improved clinician practice choices [19,20].

Knowledge is one of many clinical practice barriers to modifying medication-related weight gain, and merits incorporation into future initiatives. The findings, taken with the population prevalence of obesity, the emerging treatment options, and the central role of the primary care practitioner, suggest a significant prevention opportunity.

## Conclusions

Pediatricians’ knowledge base of adverse metabolic drug effects appears comparable to their counterparts in adult medicine. Regardless of medical specialty, practitioners participating in the CME programs reflected low knowledge on specific questions pertaining to drug indications, adverse metabolic effects, patient risk profiles and safety updates. Each of the four knowledge gaps would potentially influence clinical decision-making in the same manner, leading clinicians to overestimate the benefits of a drug in relation to its metabolic risks. Therefore future efforts to detail cross-specialty practitioner knowledge of metabolic drug effects and initiate education strategies to bolster knowledge could meaningfully contribute to obesity prevention.

### Availability of supporting data

The full instrument (CME questions from all activities) is available at the journal’s request.

## Competing interests

Neither author has financial or non-financial competing interests.

## Authors’ contributions

IK developed the CME modules in collaboration with colleagues acknowledged elsewhere in the manuscript and published CME materials. She designed the study in collaboration with the Office of Pediatric Therapeutics and drafted the manuscript. GW participated in the design of the study and performed the statistical analysis. Both authors read and approved the final manuscript.

## Author’s information

IK is a physician nutrition specialist board-certified in preventive medicine and public health. She is the editor of Advancing Medicine with Food and Nutrients, Second Edition (CRC Press, December 2012) and serves on the faculty of Johns Hopkins Bloomberg School of Public Health. As an inaugural FDA Commissioner’s Fellow she worked within the Office of Pediatric Therapeutics on nutrition-related issues, which gave rise to this research collaboration.
